# Biological aging for the advancement of biogerontology: Between hopes and hypes

**DOI:** 10.1016/j.jnha.2026.100992

**Published:** 2026-09-26

**Authors:** Emanuele Marzetti, Anna Picca

**Affiliations:** a Department of Geriatrics, Orthopedics and Rheumatology, Università Cattolica del Sacro Cuore, Rome, Italy; b Fondazione Policlinico Universitario “A. Gemelli” IRCCS, Rome, Italy; c Department of Medicine and Surgery, LUM University, Casamassima, Italy

**Keywords:** Aging, Biomarkers, Geroscience, Healthspan, Intrinsic capacity, Resilience

## Abstract

Population aging has intensified the search for biological measures that can explain why individuals of the same chronological age differ so markedly in health, function, resilience, and disease burden. Geroscience has provided a powerful framework by linking shared mechanisms of aging to multiple age-related conditions, but translation to clinical practice remains incomplete. Aging clocks and circulating biomarkers have improved biological-age estimation, yet prediction alone does not establish biological meaning or clinical utility. Emerging evidence instead supports a view of aging as a heterogeneous and multidimensional process shaped by organ-specific vulnerability, cumulative stress, multimorbidity, and declining resilience. The field should therefore move beyond single biomarkers and universal aging clocks toward gerodiagnostic approaches that characterize individual aging trajectories and their modifiability. Progress in gerotherapeutics will similarly require closer integration of mechanism, measurement, and intervention. The ultimate goal is not merely to quantify aging, but to identify actionable biological processes whose modification may preserve function, delay multimorbidity, and extend healthspan.

Population aging is amongst the defining transformations of contemporary societies. Yet the remarkable extension of human lifespan achieved over the past century has not been accompanied by an equivalent increase of healthspan [1]. The resulting gap between years lived and years lived in health has profound individual, healthcare, and socioeconomic consequences. This discrepancy also highlights one of the main challenges and limitations of modern medicine: while highly successful in treating individual diseases, it remains largely organized around conditions that frequently coexist and share aging as their strongest risk factor. This concept has laid the ground for the foundation of geroscience, which proposes that targeting the biological mechanisms of aging may simultaneously delay or attenuate multiple age-related diseases and impairments [2]. Rather than considering age-associated conditions (e.g., cardiovascular disease, cancer, neurodegeneration, diabetes, physical disability) as separate entities, geroscience asks whether at least part of their increasing prevalence with advancing age originates from a shared deterioration of biological processes that constitutively preserve homeostasis and resilience.

This paradigm has revolutionized biogerontology. The description of the hallmarks of aging provided a powerful framework for organizing an extraordinarily complex biology into experimentally tractable domains [3]. Its subsequent expansion into twelve highly interconnected aging pillars further emphasized that aging cannot be reduced to a single pathway but emerges from interactions among genomic instability, telomere attrition, epigenetic alterations, loss of proteostasis, disabled macroautophagy, deregulated nutrient sensing, mitochondrial dysfunction, cellular senescence, stem cell exhaustion, altered intercellular communication, chronic inflammation, and dysbiosis [4]. The impact of this paradigm has been enormous. Yet, because of this success, the field now faces a different challenge, namely moving from describing the biology of aging to measuring, stratifying, and ultimately modifying it in humans.

## 1 From chronological age to biological aging

Chronological age remains the simplest and most robust population-level predictor of age-related outcomes. However, individuals of the same chronological age can differ dramatically in physical and cognitive function, multimorbidity, vulnerability to stressors, and residual lifespan. Some people develop multimorbidity and functional limitations relatively early, whereas others preserve remarkable physiological function into very old age. Aging is therefore universal, but its rate, biological expression, and clinical consequences are highly heterogeneous.

The distinction between chronological and biological aging emerged precisely from this observation. Biological age should ideally describe where an individual lies along an aging trajectory independent of the number of years lived. This seemingly simple concept has fueled an enormous search for biomarkers able of capturing aging more accurately than chronological time [5]. However, the challenge is not merely to identify variables that correlate with age. An informative biomarker of aging should provide information about biological deterioration, predict clinically relevant outcomes, respond to changes in the underlying aging process, and ideally help identify the mechanisms responsible for accelerated or resilient aging [6]. This distinction is essential because a predictor of chronological age is not necessarily a measure of biological aging. Epigenetic clocks exemplify both the extraordinary potential and some limitations of this approach. Early clocks were remarkably accurate in formulating chronological age from DNA methylation patterns. Yet an algorithm optimized to predict a time passage does not automatically identify the biological processes that determine future disease, disability, or resilience. Newer generations of clocks and other omics-based predictors have increasingly incorporated mortality, physiological variables, or health outcomes, improving their biological relevance [7]. Nevertheless, the proliferation of aging clocks [8] also illustrates one of the current “hypes” of the field as predictive accuracy alone does not necessarily correspond to mechanistic understanding.

## 2 From aging clocks to gerodiagnostics

The emerging field of aging biomarkers needs to move beyond the estimation of biological age toward the characterization of individual aging trajectories and the biological processes that shape their progression, clinical consequences, and potential for modification. Recent proteomic approaches illustrate this transition. Organ-specific plasma protein signatures have shown that aging does not proceed uniformly across organs within the same individual and that accelerated aging of organs can be associated with organ-specific diseases and mortality [9]. Such observations challenge the concept of a single biological age and suggest that individuals may instead possess a mosaic of biological ages, reflecting differential vulnerability and compensatory capacity across tissues.

This view is consistent with a broader concept of aging as progressive loss of resilience. Molecular damage accumulates throughout life, but its phenotypic consequences depend on the capacity of cells, tissues, and physiological systems to detect, compensate for, repair, or adapt to perturbations. Once these mechanisms become insufficient, initially silent molecular alterations may become functional deficits and, eventually, clinically recognizable disease. Intrinsic capacity (IC) offers a complementary concept through which the transition from molecular change to organism-level function may be considered. Defined as the composite of an individual’s physical and mental capacities across cognition, locomotion, vitality, psychological, and sensory domains, IC shifts the focus from accumulated deficits toward the residual individual capacities. From a geroscience perspective, IC may therefore be viewed as an organism-level expression of the balance between biological damage and resilience. Importantly, IC appears to comprise both a general component shared across domains and domain-specific variation, consistent with the notion that common age-related processes may be expressed heterogeneously across physiological systems [10]. Evidence from longitudinal studies link lower IC with functional decline and mortality [10,11], while emerging genomic data indicate a measurable genetic architecture of IC [12]. IC may thus represent a multidimensional phenotype through which the biological heterogeneity of aging may be interrogated.

The recently proposed brain–body energy conservation model provides an intriguing view of how molecular damage, cellular stress responses, systemic signals, and organismal phenotypes may be connected [13]. According to this framework, the energetic cost associated with cellular damage and stress responses generates systemic signals that may ultimately contribute to adaptive energy-conservation programs. Whether all aspects of this model translate to human aging remains to be established, but its systems-level perspective is important. Aging biology does not occur within isolated pathways: molecular damage, mitochondrial stress, inflammation, metabolism, neuroendocrine regulation, and behavior continuously interact. Such complexity argues for a transition from isolated biomarkers toward gerodiagnostics, defined as integrated measures capable of characterizing biological vulnerability, resilience, and stress responsiveness at the individual level.

Multimorbidity provides a particularly informative clinical phenotype in which to test such a proposition. A recent longitudinal cohort study identified a shared circulating biomarkers signature composed by growth differentiation factor 15 (GDF-15), glycated hemoglobin, cystatin C, leptin, and insulin, associated with overall disease burden, distinct multimorbidity patterns, and the subsequent rate of disease accumulation, while additional biomarkers showed greater phenotype specificity [14]. These findings support the existence of a common biological substrate underlying clinically heterogeneous manifestations of aging but also challenge the notion that biological aging can be adequately represented by a single biomarker or universal molecular clock. However, the distinction is more than semantic. A biomarker associated with multimorbidity is not necessarily a biomarker of biological aging. The same signal, indeed, may integrate aging mechanisms, organ dysfunction, established disease, and compensatory responses. Longitudinal phenotypes may therefore be particularly informative. A marker associated with how rapidly diseases accumulate arguably comes closer to capturing an aging trajectory than one trained simply to reproduce chronological age. Herein lies the urge of the field moving from predicting age to predicting aging. In this perspective, multimorbidity and IC provide complementary windows into the clinical expression of biological aging: the former captures the accumulation and organization of disease, whereas the latter captures the preservation or loss of functional reserve across multiple domains.

## 3 Biomarkers as biological signals, not merely predictors

The distinction between biomarkers role as biological signals or predictors becomes particularly relevant when considering circulating biomarkers. Molecules such as GDF-15, fibroblast growth factor 21 (FGF-21), inflammatory mediators, neurofilament light chain, and numerous metabolites have been associated with aging phenotypes and adverse outcomes. Their value, however, may reside not simply in their ability to predict an event, but in the biological information they carry.

GDF-15 provides an informative example. This stress-responsive cytokine rises in several conditions characterized by mitochondrial, metabolic, or inflammatory stress and is consistently associated with adverse health outcomes [15]. Recent longitudinal evidence indicates that higher circulating GDF-15 is associated not only with prevalent sarcopenia but also with an earlier trajectory of sarcopenia progression in community-dwelling older adults [16]. Such findings support the possibility that circulating stress signals may help identify individuals whose biological trajectories are diverging before advanced functional deterioration becomes evident.

GDF-15 also illustrates why biomarkers cannot be interpreted outside their biological context. Its increase may represent damage, an adaptive response to damage, or both, depending on tissue, magnitude, duration, and clinical setting. Remarkably, inhibition of GDF-15 with ponsegromab in patients with cancer cachexia and elevated GDF-15 improved body weight, activity, and cachexia-related symptoms, providing evidence that a biomarker can, in a specific context, identify a biologically actionable pathway [17]. The lesson for geroscience is important: the strongest biomarkers may ultimately be those that connect measurement to mechanism and mechanism to intervention.

This raises a central question for gerodiagnostics: does a marker such as GDF-15 measure biological aging itself, or the cumulative burden of cellular stress, metabolic dysfunction, and disease that accompanies aging? Rather than weakening its value, this ambiguity defines the validation challenge. The strongest candidates will be those for which longitudinal change can be linked to a biologically interpretable process and to clinically meaningful trajectories, rather than merely to chronological age or prevalent disease.

Mitochondrial biology offers another example. Mitochondrial dysfunction is a hallmark of aging, but mitochondria are signaling organelles under several circumstances. They participate in stress sensing, inflammatory signaling, cell death, and intercellular communication. Mitochondrial quality control mechanisms, including proteostasis, dynamics, mitophagy, and mitochondrial-derived vesicles, continuously monitor organelle integrity and determine whether damaged components are repaired, segregated, or eliminated [18,19]. When these systems become overwhelmed, mitochondrial constituents may also reach the cytosol or extracellular environment. Mitochondrial DNA, for instance, can act as a potent danger signal, linking organelle damage to innate immune activation [20].

These observations broaden the biomarker concept. Circulating mitochondrial components, extracellular vesicles, metabolites, inflammatory mediators, and stress-responsive proteins may not simply be passive correlates of aging. They may constitute a molecular language through which tissues communicate their energetic state, damage burden, and adaptive capacity. Deciphering this language could become central to precision geromedicine.

## 4 The promise and the hype of gerotherapeutics

The ultimate ambition of geroscience is not merely measuring aging but to intervene on it. Experimental manipulation of nutrient sensing, cellular senescence, autophagy, mitochondrial quality control, and other aging-related pathways has produced striking extensions of lifespan and healthspan in model organisms. These discoveries have generated considerable enthusiasm for metformin, rapamycin and rapalogs, senolytics, NAD^+^ precursors, spermidine, caloric-restriction mimetics, and other candidate gerotherapeutics.

The enthusiasm is justified but so is caution. Metformin represents perhaps the most emblematic example. Epidemiological and experimental observations suggested effects on several age-related outcomes and provided the rationale for the Targeting Aging with Metformin (TAME) initiative [21]. Importantly, TAME was conceptually transformative not because metformin was expected to be a “cure for aging,” but because it proposed testing whether an intervention targeting aging biology could delay a composite of multiple age-related outcomes. This represented an attempt to translate the geroscience hypothesis into a clinically and regulatorily testable framework.

As discussed previously [22], however, successful translation requires much more than identifying compounds that extend lifespan in experimental models. The field must define appropriate populations, intervention windows, doses, clinically meaningful endpoints, safety requirements, and regulatory pathways. It must also determine whether biological effects demonstrated in model organisms occur with comparable magnitude and direction in heterogeneous older humans.

This is where hype becomes potentially counterproductive. The language of “reversing aging”, “rejuvenation”, and universal “anti-aging” interventions risks moving faster than the evidence. Aging is not a single molecular lesion, and older adults are not biologically interchangeable. An intervention that is beneficial at one stage of aging may be ineffective, or potentially harmful, at another. Pathways that become dysregulated with age frequently also serve essential homeostatic functions. Autophagy, inflammation, nutrient sensing, mitochondrial stress responses, and cellular senescence all illustrate this biological duality. The objective should therefore not be indiscriminate suppression of the biology associated with aging, but restoration of an appropriate dynamic response to stress.

## 5 Toward precision geromedicine

The next step of biogerontology should integrate three dimensions that have too often progressed separately: mechanism, measurement, and intervention.

First, mechanistic geroscience must continue to identify the biological processes responsible for declining resilience rather than simply cataloguing molecular differences between young and old organisms. Second, biomarker research must develop measures that capture these processes longitudinally and distinguish drivers of aging from compensatory responses and consequences of disease. Third, gerotherapeutic trials should incorporate such biomarkers to establish target engagement, identify responders, define optimal intervention windows, and determine whether modification of a biological pathway translates into preservation of intrinsic capacity and function, or delayed multimorbidity.

This approach will likely require moving beyond individual molecules and universal aging clocks. Multi-omic technologies, advanced imaging, wearable-derived physiology, functional assessments, environmental exposures, and longitudinal clinical phenotyping may need to be integrated to reconstruct individualized aging trajectories. However, adding more data does not automatically produce better (gero)science. The goal should not be maximal molecular complexity, but biologically meaningful and interpretable complexity.

The framework recently proposed for biomarkers of aging emphasizes precisely the need for standardized terminology, analytical validation, biological validation, and clearly defined clinical use cases [23]. A biomarker intended to stratify risk need not satisfy the same criteria as one used to monitor response to a gerotherapeutic intervention. Likewise, a biomarker associated with mortality may be clinically useful without necessarily representing a causal mechanism of aging. Recognizing these distinctions will help separate genuine progress from inflated expectations.

## 6 From hopes and hypes to evidence

Biogerontology is experiencing an exceptional moment. Aging has moved from being considered an inevitable background condition to becoming a biologically targetable process with potentially modifiable components. This represents an extraordinary conceptual achievement. The possibility of delaying multiple age-related conditions by acting on shared biological mechanisms is one of the most compelling promises of modern biomedical science. However, the success of the field will ultimately be judged neither by the number of hallmarks identified, nor by the number of aging clocks developed, nor by the size of the longevity market. It will be judged by whether knowledge of aging biology improves human health. Achieving this goal requires a shift from biological age to biological trajectories, from biomarkers to gerodiagnostics, from associations to mechanisms, and from generic gerotherapeutics to biologically informed interventions. It also requires accepting that heterogeneity is not noise to be eliminated but one of the fundamental characteristics of human aging that must be understood. The central question for the coming decade is therefore no longer simply whether aging can be measured or manipulated. It is whether we can determine which biological processes are driving aging in a specific individual, at a specific time, and whether modifying those processes will meaningfully alter that person’s trajectory of health and function.

If geroscience can answer that question, the current hopes surrounding biological aging may become more than promises. They may provide the foundation for a new wave of preventive and precision medicine, one in which understanding the biology of aging becomes a means not merely to add years to life, but to preserve resilience, function, and health across those years.

## CRediT authorship contribution statement

**Emanuele Marzetti**: Conceptualization, Writing – original draft preparation; **Anna Picca**: Conceptualization, Supervision, Writing – review and editing.

## Declaration of Generative AI and AI-assisted technologies in the writing process

The authors used ChatGPT-5.5 to assist with language refinement. All the manuscript content has been reviewed and edited by the authors which take full responsibility for the accuracy, integrity, and originality of the work.

## Funding

This work was partially funded by the Italian Ministry of Health (Ricerca Corrente 2026), intramural research grants from the Università Cattolica del Sacro Cuore (D1.2024 and D1.2025), and Centro Studi Achilla e Linda Lorenzon (N/A).

## Declaration of competing interest

EM reports financial support was provided by Italian Ministry of Health. AP has no known competing financial interests or personal relationships that could have appeared to influence the work reported in this paper.
